# Clinical application of unilateral biportal endoscopic lumbar interbody fusion in lumbar degenerative diseases: a multi-center study

**DOI:** 10.3389/fneur.2025.1673110

**Published:** 2025-11-18

**Authors:** Yunsheng Chen, Jian Wu, Qiaoxin Li, Yaohong Wu, Canhua Xu, Rongchun Chen, Jiangyou Shi, Liping Liu, Linbo Sun, Jun Xiong

**Affiliations:** 1 Department of Spinal Surgery, Ganzhou People’s Hospital, Ganzhou, Jiangxi, China; 2 Department of Orthopedics, Shangyou County People’s Hospital, Ganzhou, Jiangxi, China; 3 Department of Orthopedics, Quannan County People’s Hospital, Ganzhou, Jiangxi, China

**Keywords:** lumbar degenerative diseases, unilateral biportal endoscopic lumbar interbody fusion, posterior lumbar interbody fusion, interbody fusion rate, inflammatory mediators, spinal function

## Abstract

**Objective:**

This study endeavors to evaluate the clinical efficacy of unilateral biportal endoscopic lumbar interbody fusion (ULIF) in managing lumbar degenerative diseases (LDD) through a multi-center investigation.

**Methods:**

One hundred patients diagnosed with LDD between August 2022 and August 2024 were enrolled and allocated to either the ULIF cohort (ULIF group, *n* = 50) or the conventional open posterior lumbar interbody fusion (PLIF) cohort (PLIF group, *n* = 50). Surgical metrics (operative time, intraoperative blood loss, postoperative drainage volume, and hospital duration) alongside the three-month interbody fusion rate were compared. Preoperative and postoperative pain Visual Analogue Scale (VAS) scores, serum inflammatory cytokine profiles (TNF-*α*, IL-6, IL-4), Japanese Orthopaedic Association (JOA) scores, and Oswestry Disability Index (ODI) scores were analyzed. Postoperative complications were documented.

**Results:**

The ULIF group demonstrated a higher three-month interbody fusion rate than the PLIF group (*P* < 0.05). Additionally, the ULIF cohort exhibited shorter operative times, diminished intraoperative blood loss, reduced postoperative drainage, and abbreviated hospital stays compared with the PLIF group (*P* < 0.05). Seven-day postoperative VAS scores were lower in the ULIF group relative to the PLIF group (*P* < 0.05). TNF-*α* and IL-6 levels were lower, while IL-4 was elevated in the ULIF cohort at 7 days postoperatively relative to the PLIF group (*P* < 0.05). JOA scores were superior and ODI scores were lower in the ULIF group at 7 days and 3 months postoperatively versus the PLIF group (*P* < 0.05). No significant difference in overall postoperative complication rates was observed between groups (*P* > 0.05).

**Conclusion:**

ULIF enhances postoperative pain relief, functional recovery, and inflammatory resolution, while simultaneously shortening operative and hospitalization durations, minimizing intraoperative blood loss, and maintaining a favorable safety profile in LDD patients.

## Introduction

Globally, lumbar degenerative diseases (LDD) stand as the primary etiology responsible for chronic lower back pain among elderly individuals (1, 2). Moreover, with the rising proportion of elderly individuals within the global population, the prevalence of these diseases continues to escalate (3). LDD encompasses a range of pathological conditions resulting from lumbar spine structural degeneration, including lumbar spinal stenosis, degenerative lumbar spondylolisthesis, lumbar disc herniation, and degenerative lumbar scoliosis (4, 5). When non-surgical therapeutic approaches, including pharmacological management and physiotherapy, fail to yield the desired therapeutic outcomes, surgical intervention frequently becomes a necessary course of action (6). Spinal fusion is widely regarded as the benchmark surgical procedure for the management of LDD in patients who exhibit a lack of response to conservative treatment modalities and experience a progression of symptoms (7).

Posterior lumbar interbody fusion (PLIF), a surgical procedure employing a posterior approach to the lumbar spine, involves dissecting muscle tissue bilaterally from the spinous process, partially removing the lamina and ligamentum flavum, and exposing the dural sac and nerve roots to achieve target disc exposure for fusion (7, 8). This surgical procedure offers several advantages, including a broad operative field of view and thorough exposure of the nerve root, achieved without compromising the blood supply to the graft via a posterior approach, as well as facilitating the possibility of achieving a 360° fusion through a single incision (7, 9). However, PLIF has several drawbacks, such as the need for extensive stripping of the paravertebral muscles, resulting in greater trauma and prolonged recovery time; paravertebral medical injuries associated with long-term muscle contraction, which may lead to severe complications; and a high incidence of degenerative changes in adjacent spinal segments, even necessitating reoperation (7, 10). Under such circumstances, there is an urgent need in the current field of LDD treatment to find a safer, more effective, and minimally invasive surgical approach.

In recent years, advancements have led to the development of the biportal endoscopic system and unilateral biportal endoscopic lumbar interbody fusion (ULIF), which integrates the benefits of traditional open surgery and minimally invasive endoscopic techniques (11, 12). Endoscopic lumbar interbody fusion, performed via either uniportal or biportal endoscopic techniques, has been explored to expedite postoperative recovery (13, 14). Unlike percutaneous uniportal full-endoscopic spinal surgery, the biportal endoscopic approach provides distinct viewing and working channels, enabling continuous fluid irrigation via two separate surgical pathways (15–17). Using two independent transmuscular channels (one for visualization, one for manipulation) enables unrestricted surgical field access and dynamic instrument handling, shortening the learning curve compared to uniportal endoscopy (18, 19). Reportedly, the biportal endoscopic approach has advanced sufficiently to be applicable in lumbar interbody fusion surgery (14, 20, 21).

It is noteworthy that during the onset and progression of LDD, inflammatory responses play a pivotal role (22). Tumor necrosis factor-alpha (TNF-*α*), interleukin-6 (IL-6), and interleukin-4 (IL-4), as important inflammatory cytokines, exert unique functions in regulating immune responses and inflammatory processes (23, 24). These three inflammatory factors represent typical cytokines in both pro-inflammatory and anti-inflammatory directions, comprehensively and accurately reflecting the internal inflammatory regulatory mechanisms of the body during the onset and progression of LDD, providing crucial information for a deeper understanding of the disease’s nature.

Given the above information, the research objective is to conduct a multi-center study to thoroughly explore the clinical application effects of ULIF in LDD and analyze the intervertebral fusion rate, surgical-related indicators, and serum inflammatory cytokine levels of patients before and after surgery, so as to compare the application value of this surgical approach with PLIF in LDD.

## Materials and methods

### Ethics statement

Ethical approval for the study was granted by the ethics committee of Ganzhou People’s Hospital, and informed consent was obtained from all participants.

### Patient cohort

A multi-center, prospective cohort study design was employed, involving 100 patients admitted to Ganzhou People’s Hospital between August 2022 and August 2024. Inclusion criteria encompassed: (1) diagnostic confirmation of LDD pathology (1); (2) failed conservative management for ≥ 3 months necessitating primary surgical intervention for LDDs; (3) adequate cognitive function and compliance; (4) complete demographic data; (5) signed informed consent. Exclusion criteria included: (1) concurrent spinal neoplasms, infections, or fractures; (2) prior lumbar surgical history; (3) severe cardiovascular/cerebrovascular comorbidities or coagulation disorders.

The patients were categorized into two groups based on the surgical approach: the ULIF group and the PLIF group (*n* = 50 each). There were no remarkable differences in the general demographic data between the two groups (*P* > 0.05; Table 1).

**Table 1:** Comparison of general data between the two groups.

Group	PLIF group (*n* = 50)	ULIF group (*n* = 50)	*t*	*P*
Male [*n* (%)]	27 (54.00)	28 (56.00)	0.040	0.841
Age (years)	60.26 ± 9.92	61.48 ± 11.86	0.558	0.578
Body mass index (kg/m^2^)	23.86 ± 2.52	23.72 ± 2.61	0.261	0.795
Disease duration (year)	5.84 ± 1.33	5.88 ± 1.48	0.142	0.887
Disease type [*n* (%)]			0.216	0.975
Lumbar disc herniation	23 (46.00)	24 (48.00)		
Lumbar spinal stenosis	14 (28.00)	13 (26.00)		
Lumbar spondylolisthesis	8 (16.00)	7 (14.00)		
Degenerative lumbar instability	5 (10.00)	6 (12.00)		
Lesion segment [*n* (%)]			0.756	0.685
L3 ~ L4	4 (8.00)	2 (4.00)		
L4 ~ L5	40 (80.00)	41 (82.00)		
L5 ~ S1	6 (12.00)	7 (14.00)		
Number of cases in each center			3.669	0.160
Center 1 (*n* = 35)	20 (40.00)	15 (30.00)		
Center 2 (*n* = 32)	18 (36.00)	14 (28.00)		
Center 3 (*n* = 33)	12 (24.00)	21 (42.00)		

### Randomization method and blinding method

The 100 finally included patients were numbered from 1 to 100 according to the order of enrollment. Corresponding numbers of random numbers were extracted from the “Random Number Table.” After arranging these numbers in ascending order, the first 50 patients were assigned to the PLIF group, and the latter 50 patients were assigned to the ULIF group. The grouping scheme was prepared and sealed by an independent statistician, and it was unsealed by the researchers before the implementation of the intervention. As an open-label clinical trial, both the patients and clinicians were aware of the treatment methods they would receive, and patients were required to cooperate with doctors or therapists before treatment. The researchers responsible for the postoperative clinical efficacy assessment were blinded to the grouping. During the data collection and analysis phases, clinical researchers, assessors, and statisticians did not share research information with each other.

### Surgical techniques

Both groups underwent preoperative evaluation and general anesthesia with prone positioning.

#### The PLIF group

Conventional PLIF was performed (25). A midline incision was made along the lumbar spine, followed by sequential dissection through the skin and lumbar fascia. Paraspinal muscles were bluntly separated while preserving the bilateral facet joint structures. Intraoperatively, the PLX118WF C-arm X-ray system (Nanjing Puao Medical) was used for imaging guidance to accurately place the pedicle screw system. Subsequently, ligamentous tissues, spinous processes, and bilateral laminae in the affected region were removed. Posterior scar tissue within the intervertebral space was also excised, and decompression was performed to enlarge the nerve root canal and lateral recess while preserving facet joint integrity. Upon completion of the main procedure, the surgical field was irrigated, hemostasis was achieved, and layered closure was performed. A drainage device was routinely placed. For cases with confirmed severe lateral recess stenosis, additional steps were taken: the diseased intervertebral disc and nucleus pulposus were removed, cartilaginous endplates were thoroughly cleared using a curette, and nerve root decompression was ensured. Finally, an autologous bone graft and an interbody fusion cage were implanted to complete stabilization.

#### The ULIF group

ULIF approach was utilized (26), with key procedural steps as follows.

After general anesthesia, the patient was placed in a prone position. The responsible intervertebral space was located under fluoroscopic guidance using a C-arm X-ray machine, and the surface projections of the upper and lower pedicles were marked. Under fluoroscopic guidance, four positioning guidewires were implanted through the pedicles. Two transverse incisions were made on the side with more severe symptoms: a proximal incision (approximately 1 cm in length) served as the observation channel, and a distal incision (approximately 2 cm in length) served as the operative channel. An arthroscope was inserted, and surrounding soft tissues were cleared. After hemostasis with a radiofrequency probe, the lower edge of the lamina of the superior vertebra and the upper edge of the lamina of the inferior vertebra were sequentially exposed. Part of the lower edge of the superior lamina, the facet joint, and the ligamentum flavum were removed to expose the dural sac and nerve root. After retracting the nerve root with a nerve hook, the intervertebral nucleus pulposus tissue was removed using nucleus pulposus forceps and reamers. Further treatment was carried out on the ipsilateral lamina and the medial part of the facet joint. The nerve root canal and lateral recess were enlarged with a lamina rongeur to achieve adequate decompression.

After scraping the endplates, a mixture of autologous and allogeneic bone graft particles was implanted. A cage was inserted and initially distracted and fixed. After confirming the position under C-arm fluoroscopy, four pedicle screws were routinely implanted (in cases of lumbar spondylolisthesis, the intervertebral space needed to be fully treated before screw insertion). On the contralateral side of the cage, connecting rods and set screws were installed. After confirming satisfactory reduction and fixation under fluoroscopy, all set screws were tightened to complete the final fixation. After checking for no active bleeding, a drainage tube was placed, and the skin incision was sutured layer by layer. Postoperative treatment was the same as that in the control group. All cases were performed by a fixed surgical team, and postoperative anti-infection and rehabilitation guidance were uniformly provided. Patients were followed up for 3 months through telephone or outpatient follow-up.

### Observation indicators

1. Interbody fusion rate: At 3 months postoperatively, interbody fusion was assessed using the Bridwell grading system (27). Grade I indicated definite bony fusion with continuous trabecular bone bridging the intervertebral space; Grade II indicated incomplete fusion without radiolucent lines and no implant loosening; Grade III indicated presence of radiolucent lines or implant loosening without collapse; Grade IV indicated fusion failure with implant displacement or collapse. Grades I and II were considered successful fusion, while Grades III and IV were defined as nonunion. The fusion success rate was calculated based on assessments conducted by two independent radiologists in a blinded manner, using dynamic lumbar X-rays and 1-mm slice thickness CT reconstructions at 3 months postoperatively. In cases of disagreement, a senior radiologist adjudicated the final evaluation.
2. Surgical metrics: Operative time, intraoperative blood loss, postoperative drainage volume, and hospital duration were compared in two groups.
3. Visual Analogue Scale (VAS) scores: Preoperative and seven-day postoperative VAS scores were compared (28). Patients self-reported pain intensity on a 10 cm scale, translated to numerical scores (0–10), with higher scores for greater pain.
4. Serum inflammatory cytokine profiles: Standard venous blood sampling technique was employed preoperatively and 7 days postoperatively to collect 5 mL of peripheral venous blood from each subject in both groups under fasting conditions in the morning. Immediately after collection, the samples were centrifuged at 3,000 rpm for 20 min to obtain serum. The supernatant was aliquoted and stored at −20 °C for subsequent analysis. The concentrations of serum inflammatory cytokines (TNF-*α*, IL-6, and IL-4) were measured using ELISA, with the experimental procedures strictly adhering to the kit instructions provided by Shuangying Biotechnology (Shanghai, China).
5. Japanese Orthopaedic Association Score (JOA): Preoperative, seven-day, and three-month JOA scores were compared (29). The JOA scale evaluates subjective symptoms (0–9), clinical signs (0–6), and activity limitations (0–14), with higher scores indicating superior function in a range of 0 to 29.
6. Oswestry Disability Index (ODI) scores: Preoperative, seven-day, and three-month ODI scores were compared (30). The ODI evaluates functional limitation across 10 domains of daily activity—such as lifting, walking, and sitting—with each item scored from 0 to 5, yielding a total score of 50. Functional status was stratified as follows: < 5 points indicated no disability; 5–14, mild impairment; 15–24, moderate impairment; 25–34, severe impairment; and ≥ 35, complete loss of function. Higher scores reflect greater functional limitation.
7. Safety profile: Postoperative complications (incisional infection, hematoma compression, cerebrospinal fluid leakage, and lower limb numbness) were recorded and compared between groups.

### Statistical analysis

Statistical analyses were performed using SPSS version 26.0. Normality of continuous variables was assessed using the Shapiro–Wilk test for sample sizes ≤50 or the Kolmogorov–Smirnov test for larger samples, with *P* > 0.10 indicating a normal distribution. Data conforming to normality were expressed as mean ± standard deviation ( $\bar{x\;}$ ± s). Between-group comparisons were conducted using the independent samples *t*-test (for equal variances) or Welch’s t-test (for unequal variances), while within-group comparisons used the paired t-test. For non-normally distributed data, values were expressed as median and interquartile range [M (P25, P75)], with Mann–Whitney *U* tests used for between-group comparisons and Wilcoxon signed-rank tests for within-group comparisons. Kappa Consistency Test was performed on the assessment results of the two observers. A Kappa value of 0–0.2 indicates slight agreement, 0.21–0.40 indicates fair agreement, 0.41–0.60 indicates moderate agreement, 0.61–0.80 indicates substantial agreement, and ≥0.81 indicates almost perfect agreement. Repeated measures—such as preoperative, 7-day, and 3-month postoperative data—were analyzed using repeated measures ANOVA (for normally distributed data) or the Friedman test (for non-normal data). Categorical variables were presented as counts and percentages [*n* (%)], and compared using the chi-square test or Fisher’s exact test where expected frequencies were < 5. A two-tailed *P* value < 0.05 was considered statistically significant, with *α* set at 0.05.

## Results

### General data

As shown in Table 1, there were no statistically significant differences in general information such as gender, age, body mass index, disease duration, and disease type between the two groups, indicating comparability (*P* > 0.05).

### Interbody fusion rate

At the three-month postoperative assessment, the ULIF group exhibited a significantly higher rate of successful interbody fusion compared to the PLIF group (49/50 vs. 43/50) (*P* < 0.05). The inter-observer reliability Kappa value was 0.703, indicating substantial agreement (Table 2).

**Table 2:** Comparison of interbody fusion rate between the two groups [*n* (%)].

Group	I	II	III	IV	Successful outcomes
ULIF group (*n* = 50)	39 (78.00)	10 (20.00)	1 (2.00)	0 (0.00)	49 (98.00)
PLIF group (*n* = 50)	34 (68.00)	9 (18.00)	5 (10.00)	2 (4.00)	43 (86.00)
*X^2^*					4.891
*P*					0.027

### Surgical metrics

The ULIF group demonstrated significantly shorter operative time, reduced intraoperative blood loss and postoperative drainage, and a decreased length of hospital stay compared to the PLIF group (*P* < 0.05; Table 3).

**Table 3:** Comparison of surgical metrics between the two groups ( $\bar{x\;}$ ± s).

Group	Operative time (min)	Intraoperative blood loss (mL)	Postoperative drainage volume (mL)	Hospital duration (*d*)
PLIF group (*n* = 50)	112.64 ± 14.60	87.94 ± 6.69	117.86 ± 15.01	12.60 ± 2.09
ULIF group (*n* = 50)	102.06 ± 15.26	50.94 ± 6.69	80.72 ± 17.21	8.72 ± 1.92
*t*	3.543	27.667	11.500	9.674
*P*	0.001	< 0.001	< 0.001	< 0.001

### VAS scores

Preoperative VAS scores were comparable between the two groups (*P* > 0.05). However, the ULIF group reported significantly lower VAS scores at 7 days postoperatively, indicating more rapid pain relief (*P* < 0.05; Table 4).

**Table 4:** Comparison of VAS scores between the two groups (score, $\;\bar{x}$ ± s).

Group	Before treatment	7-day after treatment
PLIF group (*n* = 50)	7.54 ± 0.71	4.54 ± 0.65^*^
ULIF group (*n* = 50)	7.36 ± 0.78	3.26 ± 0.83^*^
*t*	1.213	8.619
*P*	0.228	< 0.001

Compared to the situation before treatment, ^*^P < 0.05.

### Serum inflammatory cytokine profiles

No significant differences were observed in preoperative serum levels of TNF-*α*, IL-6, and IL-4 between the two groups (*P* > 0.05). Postoperatively, the ULIF group exhibited lower TNF-α and IL-6 levels, but higher IL-4 levels at 7 days after treatment (*P* < 0.05; Table 5).

**Table 5:** Comparison of serum inflammatory cytokine profiles between the two groups (pg/mL, $\;\bar{x}$ ± s).

Group	TNF-*α*		IL-6		IL-4	
	Before treatment	7-day after treatment	Before treatment	7-day after treatment	Before treatment	7-day after treatment
PLIF group (*n* = 50)	89.42 ± 5.15	43.78 ± 5.11^*^	232.26 ± 5.20	114.44 ± 10.37^*^	22.35 ± 3.18	31.57 ± 2.04^*^
ULIF group (*n* = 50)	89.47 ± 5.49	27.69 ± 5.36^*^	234.14 ± 5.16	89.73 ± 14.99^*^	22.03 ± 3.20	40.32 ± 3.22^*^
*t*	0.048	15.341	1.811	9.587	0.505	16.214
*P*	0.961	< 0.001	0.073	< 0.001	0.615	< 0.001

Compared to the situation before treatment, ^*^P < 0.05.

### JOA scores

Preoperative JOA scores were similar between the two groups (*P* > 0.05). Postoperatively, the ULIF group demonstrated higher JOA scores at both 7 days and 3 months (*P* < 0.05; Table 6).

**Table 6:** Comparison of JOA scores between the two groups (score, $\;\bar{x}$ ± s).

Group	Before treatment	7-day after treatment	3-month after treatment
PLIF group (*n* = 50)	9.56 ± 1.47	12.38 ± 1.56②	15.94 ± 2.82②③
ULIF group (*n* = 50)	9.78 ± 1.56	15.39 ± 2.38①②	20.88 ± 1.48①②③
*F*	1209.651 (time), 90.667 (between groups), 97.404 (interaction)		
*P*	*<*0.001 (time), <0.001 (between groups), <0.001 (interaction)		

① Compared with the PLIF group at the same time, *P* < 0.05. ② Compared with the same group before treatment, *P* < 0.05. ③ Compared with the same group 7-day after treatment, *P* < 0.05.

### ODI scores

Preoperative ODI scores were comparable between the two groups (*P* > 0.05). However, the ULIF group exhibited lower ODI scores at 7 days and 3 months postoperatively (*P* < 0.05; Table 7).

**Table 7:** Comparison of ODI scores between the two groups (score, $\;\bar{x}$ ± s).

Group	Before treatment	7-day after treatment	3-month after treatment
PLIF group (*n* = 50)	36.54 ± 3.40	26.38 ± 3.46②	17.14 ± 3.24②③
ULIF group (*n* = 50)	35.92 ± 3.51	21.68 ± 3.47①②	10.60 ± 4.19①②③
*F*	7343.500 (time), 35.568 (between groups), 171.087 (interaction)		
*P*	*<*0.001 (time), <0.001 (between groups), <0.001 (interaction)		

① Compared with the PLIF group at the same time, *P* < 0.05. ② Compared with the same group before treatment, *P* < 0.05. ③ Compared with the same group 7-day after treatment, *P* < 0.05.

### Postoperative complication rate

The overall incidence of postoperative complications was 6.00% in the ULIF group and 14.00% in the PLIF group, with no significant difference observed (*P* > 0.05; Table 8).

**Table 8:** Comparison of incidence of postoperative complication rate between the two groups (%).

Group	Incision infection	Hematoma compression	Cerebrospinal fluid leak	Lower limb numbness	Overall incidence
PLIF group (*n* = 50)	2 (4.00)	2 (4.00)	0 (0.00)	3 (6.00)	7 (14.00)
ULIF group (*n* = 50)	1 (2.00)	1 (2.00)	0 (0.00)	1 (2.00)	3 (6.00)
*X^2^*					1.778
*P*					0.182

## Discussion

In this study, we compared ULIF with conventional PLIF for treating LDD. Our results show that ULIF offers multiple clinical benefits.

ULIF improved surgical efficiency and preserved tissue. The endoscopic approach enables surgeons to perform the procedure with reduced procedural duration and minimized intraoperative blood loss. This is likely because the biportal system provides a clear view and precise access, reducing muscle and tissue damage. A previous study supports this, showing that biportal endoscopy allows for adequate nerve decompression and ligamentum flavum removal with minimal bone cutting (31). Consequently, patients in the ULIF group experience shorter hospital stays and improved early postoperative mobility, indicating accelerated functional recovery.

The recovery trajectories of ULIF-treated patients also show marked improvements. Faster pain resolution and enhanced functional recovery are observed, which can be attributed to the reduced soft tissue injury and preserved musculature integrity associated with the endoscopic technique. This finding agrees with a systematic review reporting that endoscopic spine techniques reduce postoperative pain and enable earlier walking due to smaller incisions and less muscle retraction (13). Another clinical study also found that biportal endoscopic discectomy leads to faster recovery and lower pain scores than open surgery (14). Furthermore, ULIF demonstrates a favorable inflammatory modulation profile, suggesting an attenuated surgical stress response, which echoes with study showcasing the biportal endoscopic technique elicits a lower systemic inflammatory response compared to conventional procedures (12). In this study, the specific manifestations of serum inflammatory responses were that the levels of TNF-*α* and IL-6 in the ULIF group 7 days after surgery were lower than those in the PLIF group, while the level of IL-4 was higher. TNF-α and IL-6 are typical pro-inflammatory cytokines (32), and their decreased levels suggest that ULIF may more effectively suppress post-operative inflammatory responses and reduce tissue damage caused by inflammation. IL-4, on the other hand, is a cytokine with anti-inflammatory and immunomodulatory effects (33). The elevated level of IL-4 in the ULIF group may imply that ULIF has a unique mechanism in post-operative inflammatory regulation, possibly promoting the resolution of inflammation and tissue repair by regulating the function of immune cells, thereby having a positive impact on post-operative recovery. These findings highlight the immunological benefits of minimizing soft tissue trauma, which may help reduce post-operative complications and improve healing. However, further in-depth research is needed to clarify the specific reasons for the elevated IL-4 levels in the ULIF group and their precise relationship with post-operative recovery and inflammatory regulation.

While the overall complication rates between ULIF and PLIF do not show a statistical difference, ULIF demonstrates consistent trends toward reduced adverse events across all measured categories. This finding acts in conformity with the same research depicting no statistically significant difference in the overall surgical complication rate between the ULIF and PLIF groups (12). This suggests potential safety benefits of the ULIF, particularly in reducing complications associated with open exposure, which warrants further investigation in larger prospective trials.

Radiographic outcomes reveal comparable fusion rates between the two groups, with ULIF achieving a high level of fusion efficacy while demonstrating superiority in all other evaluated parameters. Assessing the fusion rate is crucial for patients who have undergone lumbar interbody fusion, as non-union may compromise surgical outcomes and quality of life (34). The same finding is revealed in the research showing comparable fusion rates between ULIF and conventional procedure (ULIF vs. PLIF: 94.3% vs. 90.3%). Certain advantages of the biportal endoscopic system may contribute to enhancing the fusion rate of ULIF (12). The high fusion rate of ULIF, combined with its other benefits, makes it a strong alternative to open surgery, especially for patients seeking a quicker recovery with less trauma.

Despite its obvious advantages, ULIF also has potential drawbacks. Technically, this surgical approach imposes extremely stringent requirements on the operator’s skills, necessitating proficiency in dual-channel endoscopic techniques. Any slight mistake may lead to poor surgical outcomes or even severe complications such as nerve and vascular injuries (35, 36). In terms of the learning curve, ULIF is relatively steep, requiring doctors to invest more time and effort to master it. From theoretical learning to simulation training and then to clinical practice, each stage demands a substantial amount of practical experience. During the initial unskilled phase, it directly leads to prolonged surgical time and increased blood loss, affecting patient prognosis (31, 36, 37). Economically, the high-definition endoscopic system and specialized instruments required for ULIF are expensive, increasing the cost of surgery. This not only imposes a heavier financial burden on patients but also restricts the promotion and application of this technology in primary hospitals.

In conclusion, the findings of this study advocate for ULIF as a transformative approach in spinal surgery. It offers measurable benefits in surgical precision, recovery acceleration, inflammatory control, and potential safety enhancements, all of which contribute to the optimal management of LDD. However, this study also has some limitations, such as a relatively short follow-up period, which may not allow for the assessment of the long-term stability of ULIF in LDD; and a moderate sample size with limited representativeness of multi-center data, which may introduce potential bias. Future research should conduct large-scale multi-center studies with long-term follow-up. Additionally, efforts should be made to optimize and innovate the technology (e.g., using VR/AR to simulate surgical operations, shortening the learning curve for surgeons, and enhancing surgical safety). To address the issue of high equipment costs, research should be conducted on simplified ULIF techniques to lower the threshold for implementation in primary hospitals.

## Data Availability

The raw data supporting the conclusions of this article will be made available by the authors, without undue reservation.
